# Supplementary material for: Phenotypic Characteristics and Occurrence Basis of Leaf Necrotic Spots in Response of Weedy Rice to Imazethapyr
Source: Plants (Basel). 2024 Apr 28;13(9):1218. doi: 10.3390/plants13091218 (PMC11085574; doi:10.3390/plants13091218)
Supplement: Supplementary file 1 [file plants-13-01218-s001.zip › plants-2942126-supplementary.pdf]

Supplementary Tables

Table S1 Response type of weedy rice population to imazethapyr

Germplasm name	Country	First week	Second week
China lu	China	0	3
Jainaengmi 2	South Korea	2	1
Maejeonaengmi 6	South Korea	0	3
Anhui red rice 85-22	China	0	0
Anhui red rice 85-23	China	2	1
W1713	Brazil	0	1
BT6c	Bhutan	0	1
Namweonaengmi 2	South Korea	0	2
Burma47	Laos	0	1
Losanos 7	South Korea	0	1
Pangasinan 1	South Korea	0	3
Dancheon 1	South Korea	2	1
Geumtap	South Korea	2	3
Seongju 8	South Korea	0	3
Small seed	South Korea	0	0
Big seed	South Korea	0	1
Bynamanar-w3	Myanmar	0	0
K31	South Korea	2	3
07-76	China heilongjiang	2	3
07-78	China heilongjiang	0	1
07-90	China heilongjiang	0	1
LZ85	China jili	0	1
LZ87	China jili	0	1
LZ53	China laoning	0	1
LZ51	China laoning	0	3
SZ3	China jiangsu	0	0
SZ11	China jiangsu	2	1
SZ12	China jiangsu	0	3
SZ15	China jiangsu	0	1
SZ43	China jiangsu	2	1
YZ3	China guangdong	0	3
YZ14	China guangdong	0	1
0001F	China hainan	0	2
GX	China guangxi	2	2
Cheongdoaengmi 1	South Korea	0	3
Gyeongjuaengmi 1	South Korea	0	3
Uljuaengmi 1	South Korea	0	3
Gwangyangaengmi 1	South Korea	0	3
Suncheonaengmi 1	South Korea	0	3
Gwangsanaengmi 1	South Korea	3	3
Chinglepet 5	Indian	2	3
Kanyakumari 29	Indian	0	1
Kanyakumari 5	Indian	0	3
Kayathae 29	Indian	0	3
Maduras 10	Indian	0	1
Tanjore 14	Indian	0	3

vietnam30	Vietnam	0	3
vietnam46	Vietnam	1	1
vietnam53	Vietnam	0	3
vietnam92	Vietnam	0	1
vietnam147	Vietnam	0	3
vietnam157	Vietnam	0	1
vietnam193	Vietnam	0	1
Cambodia19	Cambodia	0	1
Cambodia31	Cambodia	0	3
Cambodia37	Cambodia	0	3
Cambodia42	Cambodia	0	3
Talac 19	Philippines	0	1
Nepal 27	Nepal	1	1
Burma47	Laos	0	3
Nepal E9	Nepal	0	3
C9575	Japan	0	3
C9576	Japan	0	3
W1713	Brazil	0	1
W1714	Brazil	2	1
US1	USA	2	3
US2	USA	1	3
Ch79-1	China	0	3
Ch80-2	China	0	2
heidiaogu	China	2	1
85-27	China	2	0
TKN7-3	Nepal	0	3
BT6c	Bhutan	0	1
WR-01	Sri Lanka	0	3
WR-02	Sri Lanka	0	3
WR-03	Sri Lanka	0	3
WR-04	Sri Lanka	2	1
Lao1	Sri Lanka	0	1
Za2-2	Sri Lanka	2	0
Za2-3	Sri Lanka	0	3
Za2-4	Sri Lanka	0	3
Za2-5	Sri Lanka	0	1
Za2-6	Sri Lanka	2	1
Za2-7	Sri Lanka	2	1
Za4	Sri Lanka	2	3
Za4-1	Sri Lanka	0	1
Za4-2	Sri Lanka	0	1
Za4-3	Sri Lanka	1	0
Za4-4	Sri Lanka	0	1
Za4-5	Sri Lanka	0	3
Za4-6	Sri Lanka	1	3
Za4-7	Sri Lanka	0	3
Za5-1	Sri Lanka	3	1
Za5-2	Sri Lanka	3	3
Za6-1	Sri Lanka	3	3
Za7-1	Sri Lanka	0	1

Ssalsshre-2	South Korea	0	1
Ch80-2	China	0	1
Tatkone-3	Myanmar	3	1
Myittha-4	Myanmar	1	3
Htantepm-w1	Myanmar	0	2
Htantepm-w2	Myanmar	0	3
Htanmaner-w1	Myanmar	2	1
Nyaunglaybyin-w1	Myanmar	2	3
Nyaunglaybyin-2	Myanmar	2	3
Htantepin-14	Myanmar	2	1
Htantepin-13	Myanmar	0	1
Htantepin-6	Myanmar	0	3
China black shatter-1	China	0	1
China black shatter-2	China	0	1
Bg871	Sri Lanka	0	3
Bg364	Sri Lanka	2	1
Sanno	South Korea	2	3

Note: 0 means weedy rice keep green; 1 lose green and leaf tip with little withered; 2 placing necrosis spot; 3 mixed with 1 and 2, 2 become to 1 because of withered part and necrosis part merged together. There were necrotic spot type germplasm materials in most areas, which indicated that necrotic spot type did not have regional specificity (related content at section 2.1)

Table S2 Correlation of indicators with herbicide concentration and time

Correlation coefficient	Fv/Fm	Fv'/Fm'	Y_{NO}	Y_{NPQ}	Chldx	Leaf area
R	0.717	0.672	0.683	0.344	0.687	0.672

Note: the correlation of Fv/Fm and treatment concentration is the strongest competes with other fluorescence index (related content at section 2.2.2)

Table S3 GESA enrichment analysis of necrotic materials between IMI and CK

KEGG Pathway Term Desc	Original size	ES	NES	RANK AT MAX
Protein processing in endoplasmic reticulum	374	0.51	2.11	8777
Phagosome	136	0.51	1.93	9167
Proteasome	88	0.54	1.92	8839
SNARE interactions in vesicular transport	51	0.55	1.79	10087
Biosynthesis of nucleotide sugars	142	0.46	1.76	5639
Endocytosis	330	0.42	1.74	9237
Butanoate metabolism	41	0.56	1.74	6996
Cutin, suberine and wax biosynthesis	88	0.49	1.68	6553
Flavonoid biosynthesis	140	0.44	1.68	7706
Valine, leucine and isoleucine degradation	97	0.44	1.62	8808
Stilbenoid, diarylheptanoid and gingerol biosynthesis	87	0.50	1.61	6430

Amino sugar and nucleotide sugar metabolism	452	0.38	1.60	7375

Note: According to the sorting of gene sets, the first several were related to membrane activity, indicating that the intracellular lipid activity became active and chaotic after the treatment of imazethapyr, and the programmed death pathway began to respond. (contented in section 2.3)

Table S4 Sequences of the qPCR primers

Gene symbols	Locus ID	Primer sequences
Actin	None	F-CAGCCACACTGTCCCCATCTA<br>R-AGCAAGGTCGAGACGAAGGA
OsHsp16.9A	Os01g0136100	F-CCAAGGTGGACCAGGTGAAG<br>R-ACCGATCACATCTTCAGGAGC
OsHsp16.9B	Os01g0136200	F-CCAAGGTGGACCAGGTGAAG<br>R-CCGAGTGAAACACCGCAAAC
OsHsp18.0	Os01g0184100	F-GGCAAGTTCATGCGCAAGTT<br>R-GGAATCTCATCACGCGACCT
OsCab2R	Os01g0600900	F-CGTCTCTGCAGGAGAGTGTG<br>R-ACTCGTACATCCATCCACGC
OsEFA27	Os04g0511200	F-GCATGTGGCTTTCTTCGACC<br>R-TGACCGGCATAAACCTTCCC
OsDER1	Os05g0187800	F-CAGAATAGGTGTGCAGGCGA<br>R-TTGCATCTCCCAGATAGGCG
OsAGPL3	Os05g0580000	F-GGAAGACGGATGATCGAGAAAG<br>R-CACATGAGATGCACCAACGA
OsHsfB2c	Os09g0526600	F-TGCCCCTCCCCTACACTATT<br>R-AGGCAGACGGATTACAAGGC

Note: the transcriptome validation (related content at section 2.3)

Table S5 Variance analysis of the Fv/Fm with intense light and high temperature

Source	Sum of squares	DF	Mean square	F value	Pr>F
Model	0.034a	3	0.011	11.826	0.001
Intercept	5.327	1	5.327	5634.409	0.000
Temperature	0.017	1	0.017	17.602	0.001
Light	0.13	1	0.13	13.628	0.003
Tm*light	0.004	1	0.004	4.607	0.053
Error	0.11	12	0.001		
Total	5.372	16			
Revised total	0.045	15			

Note: R^2=0.747, revised R^2=0.684 (related content at section 2.5)

Supplementary Figures

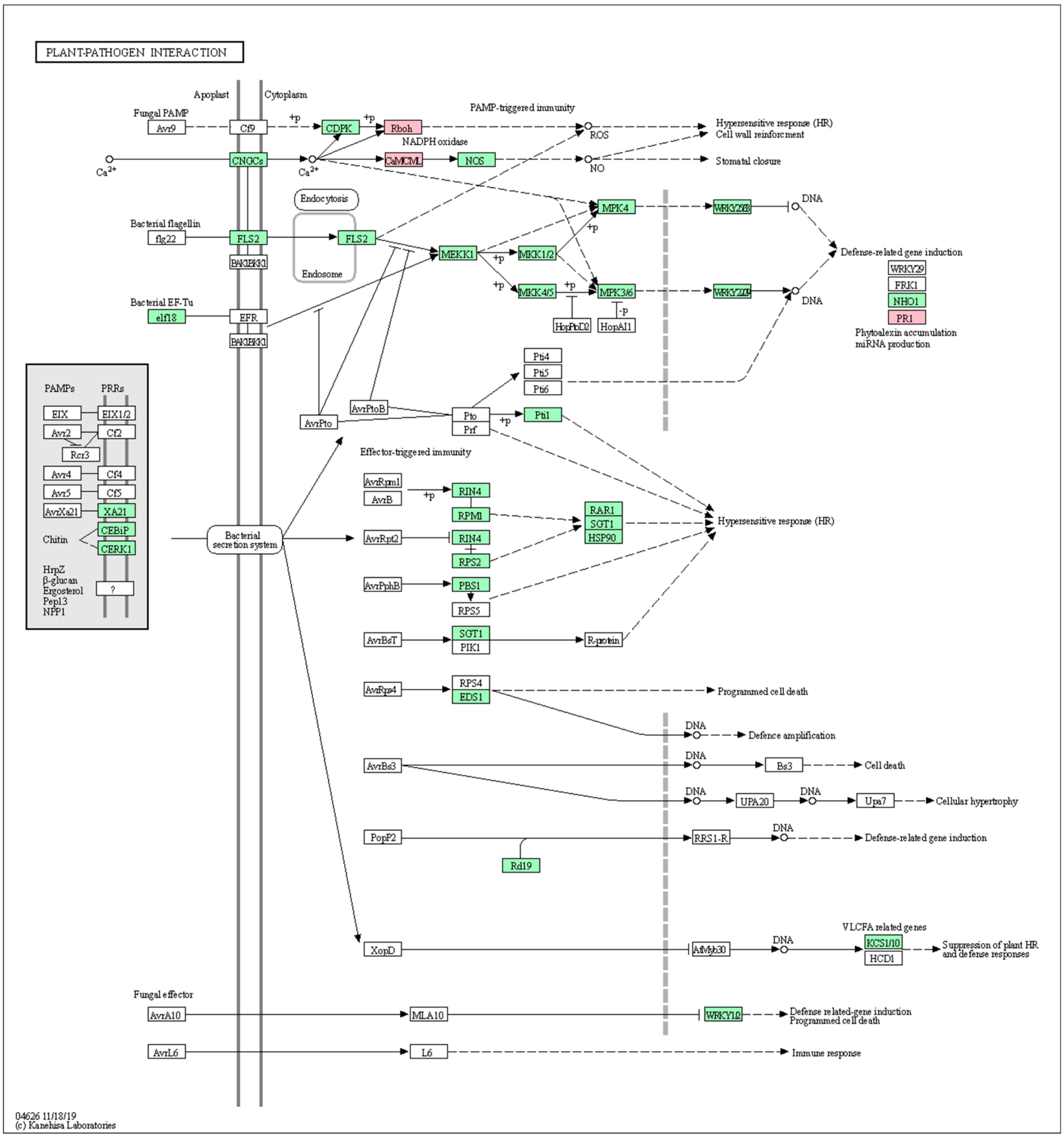

Figure S1 KEGG mapper of plant-pathogen interaction from network analysis of the top 500 different expressed genes.

Note: pink part belongs to MAPK signaling pathway.

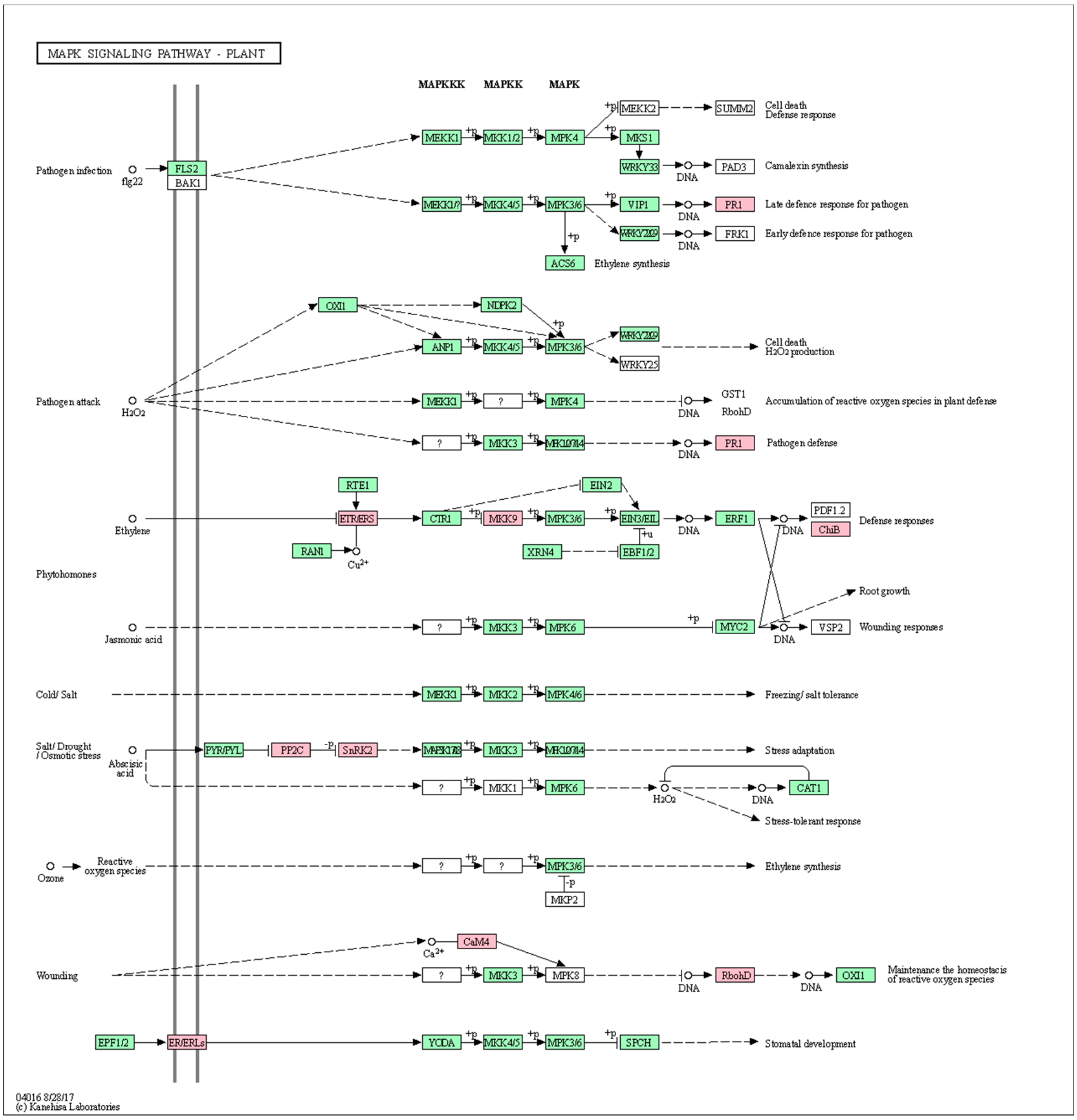

Figure S2 KEGG Mapper of the MAPK signalling pathway

Note: Pink gene selected by MAPK $\log_2$>|1| in the transcriptome.

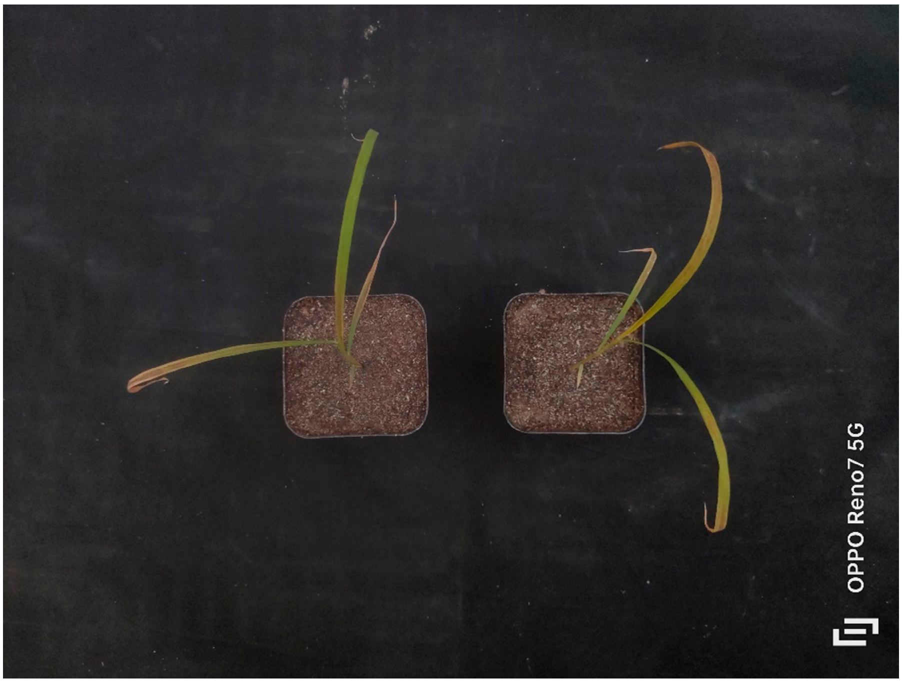

Figure S3 Comparison of herbicide damaged phenotype treated by imazethapyr and imazamox in HRT1. Note: left is imazethapyr, right is imazamox

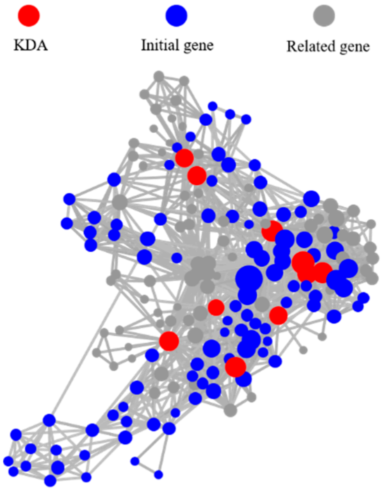

Gene ID	Log_2 Level	Description
Os02g0100100	-0.390981947	Thioredoxin domain domain containing protein.
Os02g0710900	1.753948477	Heat shock protein Hsp70 family protein.
Os03g0565100	0.643775995	OST3/OST6 family protein
Os03g0733800	1.257664078	Thiol-disulfide oxidoreductase
Os03g0832200	5.194784236	Similar to Calcium-binding protein precursor
Os03g0852200	0.360913334	Der1-like domain containing protein
Os05g0187800	1.800416693	DERLIN-like protein
Os05g0519900	0.063650552	Similar to STT3A
Os06g0644800	0.126066744	Glucosidase II beta subunit-like domain containing protein
Os07g0209000	0.641450533	Non-protein coding transcript.

Figure S4 Analysis of key driver genes in the pathway of endoplasmic reticulum stress

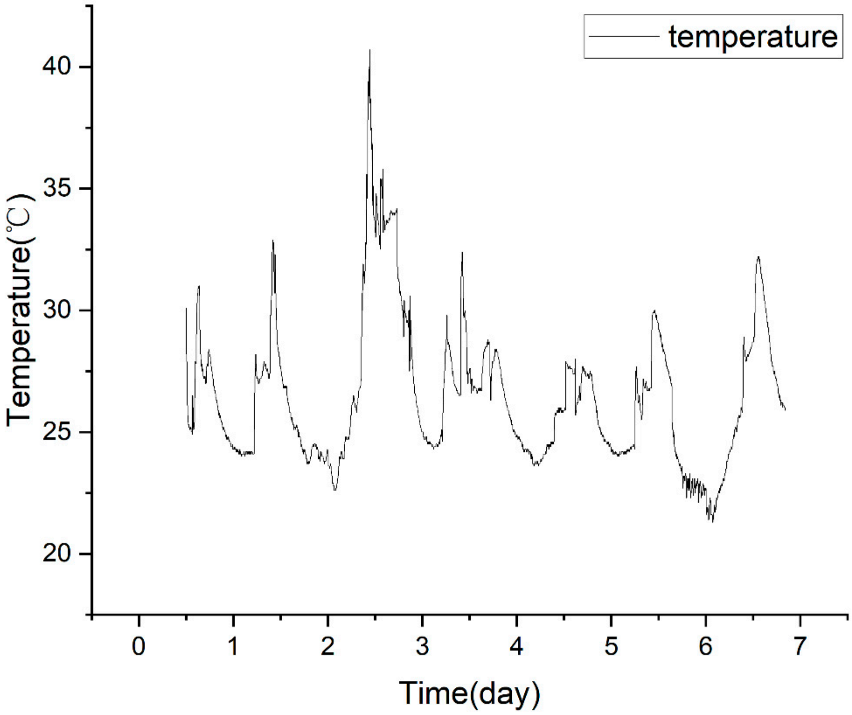

Figure S5 Temperature changes in the greenhouse used for the experiments.
